# Integrated analysis of transposon insertion sequencing and pangenome reveals core and lineage-specific essential genes in Mycobacterium avium subsp. hominissuis

**DOI:** 10.1099/mgen.0.001753

**Published:** 2026-06-25

**Authors:** Kotaro Sawai, Marie Ikai, Motoko Shinohara, Yukiko Nishiuchi, So Fujiyoshi, Yohei Doi, Tomotada Iwamoto, Kentaro Arikawa, Fumito Maruyama, Yusuke Minato

**Affiliations:** 1 Center for Infectious Disease Research, Fujita Health University, Toyoake, Aichi, Japan; 2 Departments of Microbiology and Infectious Diseases, Fujita Health University School of Medicine, Toyoake, Aichi, Japan; 3 Microbial Genomics and Ecology, Center for the Planetary Health and Innovation Science, The IDEC Institute, Hiroshima University, Higashi-Hiroshima, Hiroshima, Japan; 4 Department of Environmental and Civil Engineering, Faculty of Engineering, Toyama Prefectural University, Imizu, Toyama, Japan; 5 Center for Innovative Antimicrobial Therapy, Division of Infectious Diseases, University of Pittsburgh School of Medicine, Pittsburgh, Pennsylvania, USA; 6 Kobe Institute of Health, Kobe, Hyogo, Japan

**Keywords:** essential gene plasticity, functional genomics, *Mycobacterium avium *subsp. *hominissuis*, pan-genome, transposon insertion sequencing

## Abstract

Pulmonary disease caused by non-tuberculous mycobacteria (NTM-PD) is an emerging global health concern. Among NTM, *Mycobacterium avium* subsp. *hominissuis* (MAH) is the major causative agent of NTM-PD. Similar to *Mycobacterium tuberculosis*, MAH exhibits lineage-specific geographical distributions and host adaptations. Here, we characterized three MAH strains from the residential bathrooms of *Mycobacterium avium* complex pulmonary disease patients in Japan. A genetic population clustering analysis revealed that the three strains belong to the East Asia (EA) lineages that are predominant in Japan and Korea. Pan-genome analysis using the publicly available complete genome sequences of MAH and the newly sequenced MAH strains identified 3,313 core genes that are conserved among distinct MAH lineages. Identification of essential genes in the three strains was conducted using transposon insertion sequencing, and their gene essentiality profiles were compared to those of a previously studied sequence cluster 3 (SC3) lineage strain, MAC109. Despite their genetic diversity, nearly all essential genes were derived from the core gene set. In addition, we identified a set of common essential genes for the EA and SC3 lineages, as well as lineage-specific essential genes. Our results highlight the evolutionary and clinical importance of lineage-specific adaptations in MAH.

**Impact Statement**

By integrating transposon insertion sequencing with pan-genome analysis, we provide the first systematic comparison of essential genes across multiple *Mycobacterium avium* subsp. *hominissuis* (MAH) strains. Although MAH strains exhibit remarkable genetic diversity, we found that MAH essential genes are primarily confined to the core genome of MAH. This essential plasticity highlights the evolutionary strategies that underpin MAH survival across diverse environments and patient populations. Recognizing this interplay provides a foundation for identifying robust drug targets and developing lineage-informed therapies for MAH infection.

## Data Summary

All genome sequences have been deposited in GenBank under the following accession numbers: OCU682, chromosome AP042353 and plasmids p682a–p682c (AP042354–AP042356); OCU683, chromosome AP042357 and plasmid p683 (AP042358) and OCU803, chromosome AP042359 and plasmid p803 (AP042360). All transposon insertion sequencing and genome sequencing data are available in the NCBI BioProject database under accession number PRJDB35554, with individual Sequence Read Archive dataset accession numbers DRR704036-DRR704043 and DRR707905-DRR707908.

## Introduction

Over the past two decades, pulmonary diseases caused by non-tuberculous mycobacteria (NTM-PD), a diverse group of environmental opportunistic pathogens, have been increasingly diagnosed and reported worldwide [1,2]. The incidence of NTM-PD has risen steadily, with particularly high prevalence in East Asia (EA). Among NTM-PD, *Mycobacterium avium* complex (MAC) pulmonary disease (MAC-PD) is the most common [2,3]. For example, MAC-PD accounts for ∼90% of reported NTM-PD cases, and the incidence rate is estimated at 14.7 per 100,000 population in Japan [4].

*M. avium* subsp. *hominissuis* (MAH), the major causative agent of MAC-PD, is ubiquitous in natural and environmental sources, including soil, dust and water systems [5,6]. Recent advances in comparative genomics have highlighted the remarkable genetic diversity of MAH isolates from both clinical and environmental sources [7,9]. Based on phylogenetic and population structure analysis of MAH genomes, MAH has been divided into seven major lineages, including MAHEastAsia1 (EA1), MAHEastAsia2 (EA2) and sequence clusters (SCs) 1–5 [7,9]. Similar to *Mycobacterium tuberculosis* (*Mtb*), MAH exhibits lineage-specific geographical distributions and host adaptations, with the EA lineages being predominant in MAC-PD patients in Japan and Korea [10].

Essential genes, defined as genes indispensable for growth and/or survival, represent promising targets for the development of novel antimicrobial therapies. Transposon insertion sequencing (Tn-Seq) has emerged as a powerful tool to identify essential genes on a genome-wide scale [11,24]. Recently, Tn-Seq has been applied to identify strain/lineage-specific essential genes [13,16]. However, the application of Tn-Seq in MAH has been limited to only two strains belonging to SC3 because of the difficulty in constructing a high-density transposon mutant library [22,23]. Consequently, essential genes in non-SC3 MAH strains remain poorly understood.

Here, we sequenced the genomes of three MAH strains isolated from the residential environments of MAC-PD patients in Japan [25] and revealed that they belong to the EA1 and EA2 lineages. We generated highly saturated transposon mutant libraries from the three strains and performed genome-wide essentiality analysis using Tn-Seq. By comparing these data with previously published SC3 lineage data, we identified potential lineage-specific and core-essential genes, providing new insights into MAH biology and potential drug targets.

## Methods

### Bacterial strains, media and growth conditions

MAH strains, OCU682, OCU683 and OCU803, were isolated from MAC-PD patients' residential bathrooms [25]. MAH strains were grown aerobically at 37 °C in Middlebrook 7H9 (BD Difco^™^) medium supplemented with oleate-albumin-dextrose-catalase (OADC; 10%, vol/vol), glycerol (0.2%, vol/vol) and tyloxapol (0.05%, vol/vol).

### Whole genome sequencing by PacBio system

Genomic DNA (gDNA) was extracted from the cell pellet derived from cultured medium using the conventional phenol–chloroform method after bead beating (0.1-mm zirconia beads; Vortex Mixer GENIE2 with Microtube Attachment at max speed for 7 min) as described previously [26]. The concentrations of gDNA were measured by QuantiFluor dsDNA System on Quantus Fluorometer (Promega). The gDNA quality was confirmed by Agilent HS Genomic DNA 50 kb Kit on 5200 Fragment Analyzer System (Agilent Technologies).

Single-molecule real-time (SMRT) sequencing service provided by the Bioengineering Lab. Co., Ltd (Saitama, Japan) was used. gDNA was purified by using DNA Clean Beads (MGI Tech Co., Ltd.) and fragmented to ∼10–20 kbp using ME220 Focused-ultrasonicator (Covaris). The DNA sequencing library was prepared using the SMRTbell Express Template Prep Kit 2.0 (PacBio) following the Procedure and Checklist instructions. Polymerase complexes were prepared by Revio Polymerase kit (PacBio). Sequencing was conducted using Pacific Biosciences Revio. Among the three MAH strains, the complete genome of OCU683 was successfully assembled using PacBio Revio data.

### Additional whole-genome sequencing using Illumina and PacBio systems

For OCU682 and OCU803, additional sequencing was required to obtain complete genome assemblies. Strains were inoculated into Middlebrook 7H11 agar (BD Difco^™^) supplemented with 10% Middlebrook OADC and incubated at 37 °C for 2–3 weeks. gDNA was extracted from bacterial clumps in one inoculation loop volume (4 mm) using the conventional phenol–chloroform method after bead beating (0.2-mm glass beads; Vortex Mixer GENIE2 with Microtube Attachment at max speed for 5 min). To complete genome assemblies, OCU682 gDNA was sequenced with Illumina NovaSeq 6000 paired-end reads, whereas OCU803 gDNA was sequenced with both Illumina NovaSeq 6000 and additional long-read data from the PacBio Sequel II platform. These additional sequencing efforts were conducted at the University of Minnesota Genomics Center (Minneapolis, MN, USA).

### Whole-genome sequencing analysis

SMRT Link (ver. 12.0.0.177059) (PacBio) was used to remove overhanging sequence adaptors, and consensus sequence reads with an average quality value of less than 20 per read were removed. Filtlong (version 0.2.1) (https://github.com/rrwick/Filtlong) was used to eliminate reads shorter than 1000 bases. *De novo* assembly was performed using Flye (ver. 2.9.2-b1786) [27] and Bandage (ver. 0.8.1) [28]. The completeness of the genome was assessed using CheckM2 (ver. 1.0.1) [29]. The complete sequence of the OCU683 strain was obtained by the analysis above.

To obtain the complete sequences of OCU682 and OCU803, we conducted hybrid *de novo* genome assembly by incorporating additional Illumina sequencing data. For OCU803, additional long-read data from the PacBio Sequel II platform were also used. Hybrid assemblies were generated using Flye (ver. 2.9.2-b1801) and Unicycler (ver. 0.5.1) [30]. Genome annotation for all three strains was performed using the National Center for Biotechnology Information (NCBI) Prokaryotic Genome Annotation Pipeline (PGAP) with default settings [31].

### Lineage classification

The 185 genome sequences used for lineage classification are listed in Table S1 (available in the online Supplementary Material). Genome data for 182 strains were retrieved from the NCBI RefSeq database for entries registered as *‘Mycobacterium avium* subsp. *hominissuis’* (as of 1 May 2025). Core genome SNPs were extracted using parsnp v.2.1.1 [32], with the genome sequence of strain TH135 [33] as the reference, as described previously [7,9]. SNPs located in locally collinear blocks smaller than 200 bp, as well as sites containing gaps or ambiguous bases (N), were removed using harvesttools [34]. To determine optimal population clusters, the haplotype information of the filtered SNPs was analysed using a Bayesian hierarchical clustering algorithm in fastBAPS [35]. Visualization of the phylogenetic tree was done using iTOL [36].

### Construction of saturated transposon libraries of MAH

Saturated transposon libraries were constructed essentially as previously described [17]. The protocol was optimized by using pre-warmed buffer and phage, and maintaining continuous shaking during phage transfection for no more than 20 h. Mycobacteriophage phAE180 [37], at a titre of 2.12 to 11.0×10^10^ p.f.u. ml^−1^, was used to transduce a mariner derivative transposon Tn5371 [38] into three strains grown to an OD_600_ of 0.4–0.9. The transduced cells were resuspended in 7H9 medium, spread on a 7H9 agar plate containing tyloxapol (0.05%, vol/vol) and 50 µg ml^−1^ kanamycin and then incubated at 37 °C for 7–14 days. The resulting mutant libraries were scraped off the plates and stored at −80 °C for further gDNA extraction. Each transposon library was generated in duplicate.

### Transposon sequencing

The gDNA was extracted as described in the above section. Tn-Seq sequencing library preparation was performed essentially as previously described [39]. Briefly, 2 mM MgCl_2_ solution containing the oligonucleotides, 5′-TACCACGACCA-NH_2_ and 5′-ATGATGGCCGGTGGATTTGTGNNA NNANNNTGGTCGTGG TAT, each at 100 µM, was heated at 95 °C for 10 min and gradually cooled to 20 °C to prepare the barcoded adaptor. gDNA was fragmented using the ME220 Focused-ultrasonicator (Covaris). End repairing and ligation of the barcoded adaptors to the fragmented gDNA were performed using an NEBNext Ultra II DNA Library Prep Kit for Illumina (New England Biolabs) according to the manufacturer’s instructions. The barcoded adaptor-ligated gDNA fragments were purified with the QIAquick PCR purification kit (QIAGEN). Transposon junctions were amplified by using the transposon-specific primer T7 and the adapter-specific primer JEL_API with GoTaq colorless Master Mix (Promega) supplemented with 5% (vol/vol) DMSO under the following PCR conditions (95 °C for 10 min, 20 cycles of 95 °C for 30 s, 58 °C for 30 s, and 72 °C for 45 s, and final extension at 72 °C for 5 min). Amplification products were purified with Sera-Mag Select (Cytiva). Adaptor sequences for Illumina sequencing were added using the two-step PCR reactions below. The first PCR was performed using the Pre-Index primer mixture and Pre-Universal primer mixture with NEBNext Ultra II Q5 master mix under the following PCR conditions (98 °C for 10 min, 10 cycles of 98 °C for 10 s, 70 °C for 30 s and 72 °C for 30 s, and final extension at 72 °C for 2 min). Index PCR was performed using NEBNext Multiplex Oligos. The resultant Tn-Seq library was sequenced using a NextSeq 2000 (Illumina), 125 bp PE run using NextSeq 1000/2000 P1 XLEAP-SBS Reagents (Illumina). All primers used in this study are listed in Table S2.

### Tn-Seq analysis

Tn-Seq data were processed using the TPP tool from the TRANSIT v3.3.4 analysis platform, and transposon genome junctions were mapped to the assembled genomes of the isolate using the Burroughs–Wheeler aligner [40]. The MAC109 Tn-Seq data were obtained from the NCBI BioProject database under accession number: PRJNA527645 [22]. The MAC109 genome sequence was obtained from NCBI RefSeq and reannotated using NCBI PGAP. The reannotation was necessary in order to perform the analysis with identical procedures and parameters, thereby minimizing methodological biases and ensuring that observed differences reflected true biological variation.

Gene essentialities were assessed using the Hidden Markov Model (HMM) method provided by TRANSIT. The HMM method is based on the read count at a given site and the distribution over the surrounding sites. After the initial gene calls using the HMM method, HMM confidence scores were calculated based on the consistency of the insertion statistics with the posterior distribution of the essentiality state. Low-confidence gene calls below 0.20 were excluded. Mutual orthologues among OCU682, OCU683, OCU803 and MAC109 were defined by reciprocal best-hit analysis using blastp with an e-value cutoff of 1×10⁻¹⁰.

To assess strain-specific gene essentialities, genes identified as essential or growth defect by the HMM method were further analysed by the resampling method. For this analysis, each strain was used as a reference in separate resampling comparisons, resulting in four independent resampling analyses across the four strains. The resampling analysis compares insertion counts between strains to determine whether gene essentiality is conserved or significantly different. The *P*-values were adjusted for multiple comparisons using the Benjamini–Hochberg procedure with a false discovery rate threshold of 5%. Genes with adjusted *P*-values (*P*-adj) ≤0.05 were considered significantly different in essentiality.

### Pan-genome analysis

For the pan-genome analysis, 26 complete genomes were used (Table S1). A pairwise mash distance was calculated using Mashtree v.1.4.6 [41,42]. All genome sequences were reannotated by the NCBI PGAP. The pan-genome analysis was conducted using Roary v.3.11.2 with default parameters that identified the core, soft-core, shell and cloud genes [42]. To assess the functional categories of each gene, Clusters of Orthologous Genes (COG) assignment was performed using eggNOG-mapper v2.1.12 based on the eggNOG database, and genes were classified into COG functional categories for downstream comparative analyses [43,44].

## Results

### Lineage classification and pan-genome architecture of MAH strains

We previously isolated MAH strains from the residential bathrooms of MAC-PD patients in Japan [25]. Here, we determined the complete genome sequences of the three strains, OCU682, OCU683 and OCU803 (Table S3). The total genome sizes of the three strains ranged from 5.1 to 5.2 Mbp. Each strain harboured one to three plasmids, with plasmid sizes ranging from 17 to 151 kb. The number of predicted coding sequences ranged from 4,864 to 5,016.

**Fig. 1.**
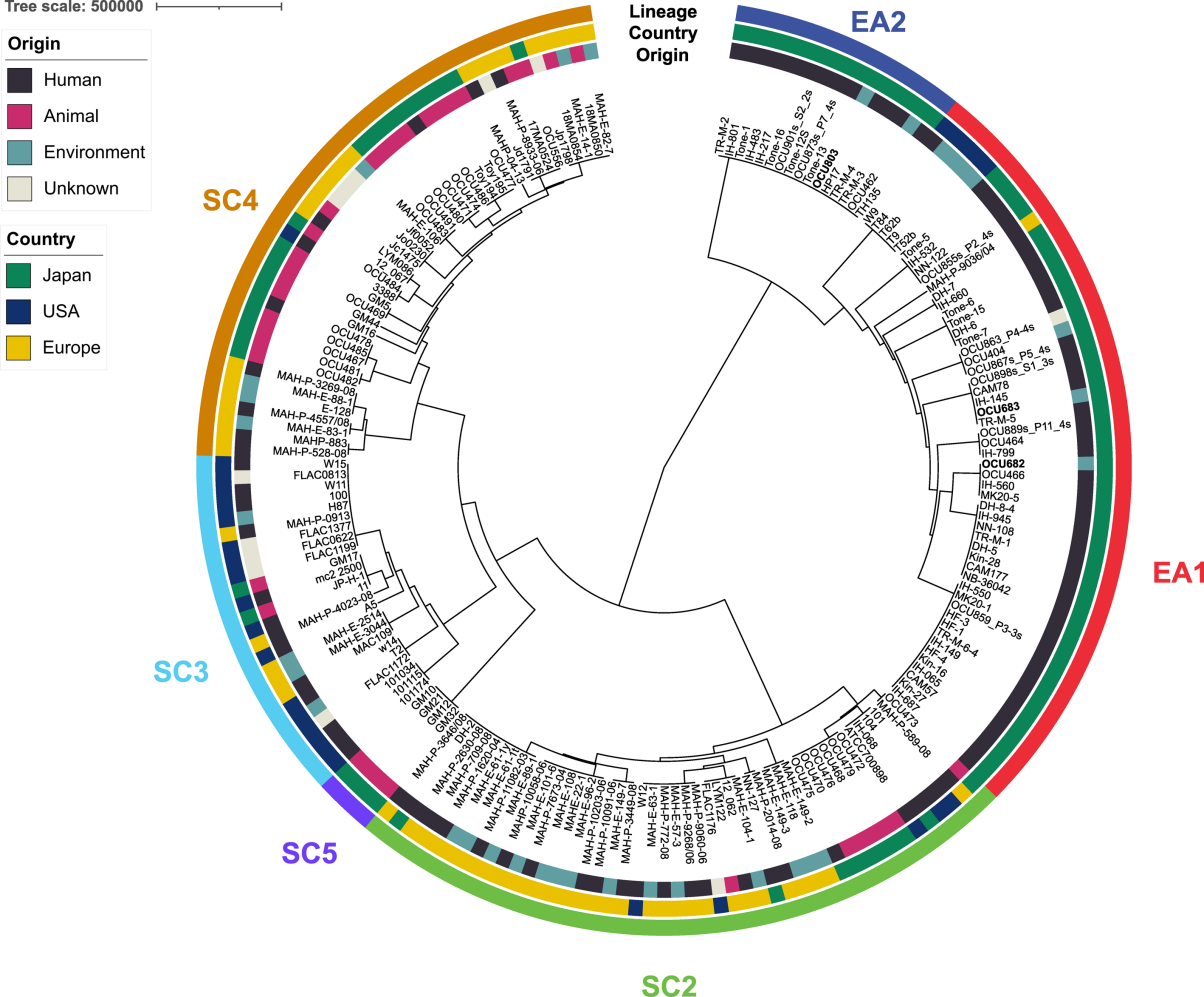
Population structure of MAH. Complete linkage clustering of 185 isolates based on SNP distances. The tree scale (branch height) represents the increase in within-cluster variance at each node under Ward’s D2 criterion. The inner ring denotes the source of isolates: black for human, magenta for animal, turquoise for environment and grey for unknown. The middle ring indicates the country of origin: green for Japan, navy for the USA and yellow for Europe. The outer ring shows lineage classifications assigned by fastBAPS: green for SC2, light blue for SC3, brown for SC4, purple for SC5, red for EA1 and navy for EA2. The isolates sequenced in this study (OCU682, OCU683, and OCU803) are highlighted with bold labels.

To determine the lineage of the three strains, we performed a genetic population clustering analysis. We searched the NCBI RefSeq database for available MAH genome sequences and obtained 182 genome sequences. Based on 41,005 core-genome SNPs, the 185 strains were classified into six lineages (Fig. 1, Table S1), consistent with previous reports [7,9]. Previously characterized 135 strains were classified into the same lineages, except for one strain, the A5 strain, which clustered into SC3 instead of SC1 [8,9] (Table S1). The most well-characterized MAH standard strain, the MAH104 strain, was classified into the SC2 lineage.

The newly analysed 50 strains were classified into five lineages: EA1, EA2, SC2, SC3 and SC4. OCU682 and OCU683 belonged to the EA1 lineage, whereas OCU803 was assigned to EA2 (Fig. 1, Table S1). The MAC109 and MAH11 strains, which were previously used to identify essential genes by Tn-Seq, were both classified into the SC3 lineage. Consistent with previous studies, strains classified into the EA1 and EA2 lineages were predominantly derived from human-associated samples in Japan, while strains classified into the SC2, SC3 and SC4 lineages were derived from various sources and regions [7,9].

### Core and accessory MAH genomes

**Fig. 2.**
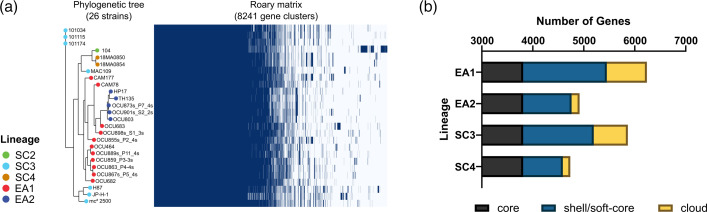
Pan-genome structure and gene content distribution across 26 MAH isolates. **(a**) The Roary matrix analysis identified 3,313 genes present in all strains. The phylogenetic tree on the left was constructed using IQ-TREE with 1,000 bootstrap replicates. (**b**) Each bar chart represents the number of core, soft-core/shell and cloud genes within each lineage. Core genes: conserved in all strains, soft-core genes: conserved in 25 strains, shell genes: conserved in 4–24 strains, cloud genes: conserved in 1–3 strains.

We next aimed to understand how many genes are conserved among different lineages. To this end, 23 complete genome sequences of MAH were obtained from the NCBI RefSeq database. We also determined the complete sequences of OCU682, OCU683 and OCU803 strains. The pan-genome analysis of the 26 strains identified a total of 8,241 unique gene clusters. Of these, 3,313 were classified as core genes (conserved in all 26 strains) (Figs 2a and S1). A core-gene-based phylogenetic tree revealed that strains from the EA1 and EA2 lineages clustered closely, while those from SC3 formed a distinct clade. The number of shell/soft-core (conserved in 4–25 strains) and cloud genes (conserved in 1–3 strains) varied across lineages (Fig. 2b). Since each isolate contains a similar number of unique genes, the total accessory gene count increases with the number of isolates within a lineage (Fig. S1). The functional composition of core and accessory genomes was broadly similar across COG categories. However, genes associated with replication, recombination and repair (COG category L) were relatively more abundant in the accessory genome (Fig. S2).

### Essential gene classification of each MAH strain using TRANSIT HMM

Because MAH strains generally show resistance to transduction by mycobacteriophage, generating a high-density transposon mutant library of MAH has been challenging [45]. To overcome this challenge, we optimized the protocol for each strain by using distinct phage titres and the length of phage transduction time. As a result, we successfully obtained high-density transposon mutant libraries for all three strains.

**Fig. 3.**
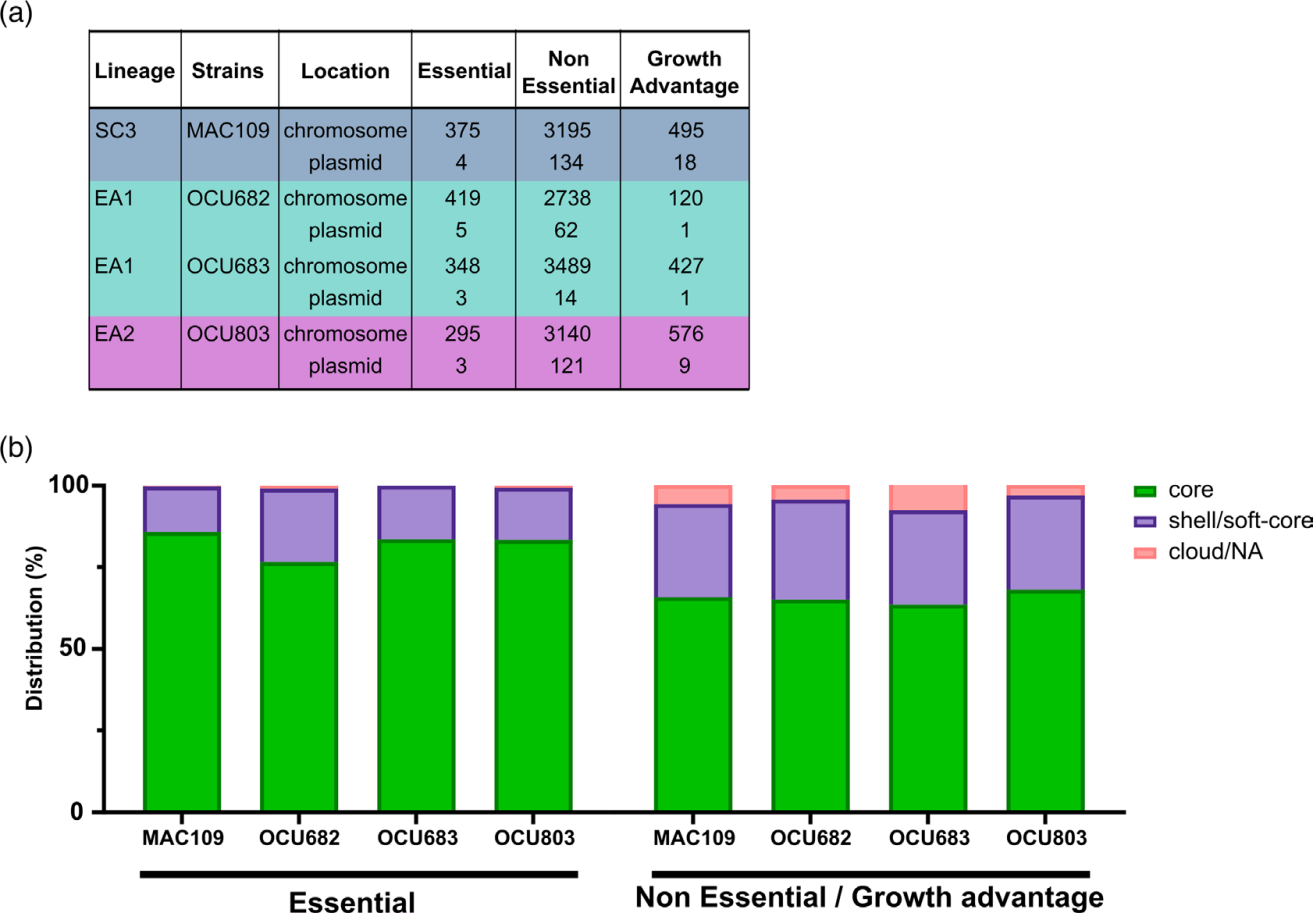
Gene essentiality classification by TRANSIT HMM analysis. (**a**) *In vitro* classification of genes in OCU682, OCU683, OCU803 and MAC109 using TRANSIT HMM. The numbers of genes classified as essential (a category that includes both essential and growth defect), non-essential and growth advantage, after excluding low-confidence genes filtered out by TRANSIT HMM analysis located on chromosomes and plasmids, are shown. (**b**) Essential genes, non-essential genes and growth-advantage genes identified by HMM analysis were mapped onto pan-genome categories defined by Roary. Genes were classified as core, soft-core, shell or cloud according to their presence across the 26 analysed *M. avium* genomes.

Genome sequencing showed that OCU682, OCU683 and OCU803 contain up to 60,667 TA dinucleotides, with an additional up to 2,536 TA sites in their plasmids. To generate saturated transposon insertion mutant libraries from each strain, we collected at least 750,000 colonies of transposon mutants from each replicate. After gDNA was extracted from the library, the transposon adjacent regions were enriched by PCR before massive parallel sequencing. The resultant sequencing data were analysed using TRANSIT software [40]. TA dinucleotides of all libraries were adequately saturated (>30% saturation) and were included in the further analysis (Table S4). Gene essentiality was assessed using the HMM method by TRANSIT. Genes identified as essential and growth defect by HMM were categorized as essential genes in Figs3  4 and S4. We identified 2,800–3,503 non-essential genes, 298–424 essential genes and 121–585 growth-advantage genes (Fig. 3a, Tables S5–S7). Almost all plasmid-encoded genes were non-essential except for several growth-defect genes and growth-advantage genes (Fig. S3, Tables S5–S7).

**Fig. 4.**
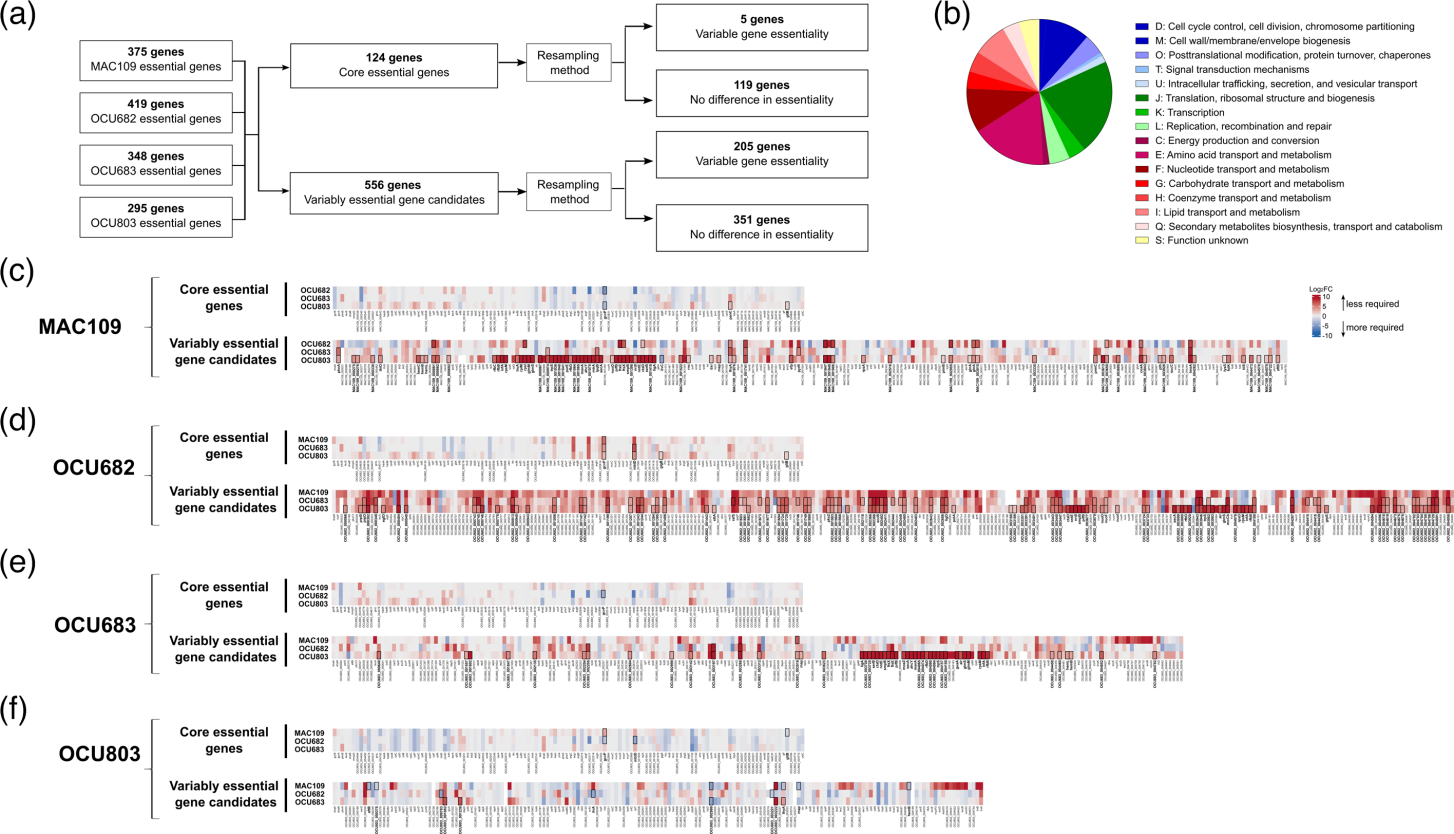
Comparison of gene essentiality among four strains by using TRANSIT resampling analysis. (**a**) Workflow of the gene essentiality comparisons. Essential genes for each strain were identified by using the TRANSIT HMM method. 124 genes were identified as core-essential genes (essential in all four strains), and 556 genes were identified as variably essential genes (one strain-specific, two strain-specific and three strain-specific genes). Both categories were further evaluated using the TRANSIT resampling method to determine whether their essentiality significantly differed between strain pairs (adjusted *P*≤0.05). (**b**) Distribution of COG functional categories among the 124 core-essential genes identified by the HMM method. (c–f) Heatmaps showing the essentiality profiles (log_2_ fold-change) of genes identified as essential by the HMM method in (**b**) MAC109, (**c**) OCU682, (**d**) OCU683 and (**e**) OCU803. For each strain, the upper panel displays the 124 core-essential genes, whereas the lower panel shows the remaining variably essential genes. Colours indicate relative differences in insertion abundance among strains. Black boxes indicate statistically significant differences in insertion abundance (adjusted *P*≤0.05). Gene names shown in bold denote genes with statistically significant differences in essentiality.

To enable direct comparison with the EA lineage datasets, MAC109 was reanalysed with the same pipeline to eliminate methodological biases [22]. Using this uniform pipeline, MAC109 contained 3,329 non-essential genes, 379 essential genes and 513 growth-advantage genes (Fig. 3a, Table S8). Almost all plasmid-encoded genes were categorized as non-essential, except for four growth-defect genes and 18 growth-advantage genes (Fig. S3, Table S8).

Essential, non-essential and growth-advantage genes identified by HMM were classified into core, shell/soft-core and cloud categories based on pan-genome analysis (Fig. 3b, Table S9). Most essential genes belonged to the core genome, whereas ∼40% of non-essential and growth-advantage genes were classified as accessory genes across all strains.

### Comparative analysis of essential genes across the four MAH strains

HMM-based essentiality classifications identified 124 core-essential genes, which are the genes essential in all four strains (Fig. S4, Table S9). These included functions related to DNA replication and repair, protein translation and central metabolic pathways. Several known antitubercular drug targets (*inhA, embAB, gyrAB, rpoB, mmpl3* and *dprE1*) were included in core-essential genes. Interestingly, the eight penicillin-binding proteins (PBPs) in MAH were not included in the core-essential genes (Table S9).

556 genes were identified as variably essential genes, including one strain-specific, two strain-specific and three strain-specific essential genes. By this analysis, we found five genes uniquely identified as essential in MAC109 and 167 genes uniquely identified as essential in EA lineages (Fig. S4). However, HMM-based essentiality classifications can be influenced by variation in TA site density, library saturation and sequencing depth. Thus, the strain-specific gene essentialities characterized by HMM may reflect technical variabilities rather than true biological divergence across strains.

To further evaluate differences in gene essentialities across strains, we applied the resampling framework implemented in TRANSIT to statistically test differences in insertion abundance (Fig. 4a–f, Tables S10–13). Almost all core-essential genes showed no change in essentiality upon resampling analysis, further validating their core essentiality. Only three genes (*panC*, *gftB* and *gcvP*) showed strain-dependent differences. Functional classification of the core-essential genes showed enrichment in class D (cell cycle control, cell division and chromosome partitioning), class J (translation, ribosomal structure and biogenesis) and class E (amino acid transport and metabolism) (Fig. 4b).

Among 556 genes identified as variably essential genes by HMM, resampling confirmed 205 genes as variably essential genes (Fig. 4a and c–f). Most confirmed variably essential genes were strain specific, and only five were lineage specific (three are SC2 specific, one is EA1 specific and one is commonly essential in EA1 and EA2 but not in SC2).

## Discussion

Despite the rising global incidence of MAH-related pulmonary disease, our understanding of MAH gene essentiality remains limited. MAH exhibits extensive genomic diversity, yet the functional implications of this diversity and the conservation of essential genes across lineages remain poorly understood. In this study, we combined Tn-Seq and whole-genome sequencing of three EA isolates with published SC3 data to perform a comparative functional genomic analysis. This dual approach enabled us to identify both core-essential genes and lineage-dependent differences in genetic requirement.

Comparison of functional categories between core and accessory genomes revealed broadly similar distributions across most COG classes. However, genes involved in replication, recombination and repair (COG category L) were relatively enriched in the accessory genome. This pattern is consistent with those reported in several bacterial pan-genome studies [46,48].

The core-essential genes identified in both EA and SC lineages highlight fundamental processes indispensable for MAH survival, including DNA replication and repair, protein translation and cell envelope biogenesis. Representative examples include genes encoding ribosomal subunits, aminoacyl-tRNA ligases, arabinosyltransferases, *fadD32* and *embB*, underscoring the central roles of genetic information processing, protein synthesis and the lipid-rich cell envelope. Conserved chaperones and proteases such as GroEL and Clp further emphasize the importance of proteostasis and stress tolerance. Resampling analysis further indicated that the essentiality of these genes was highly conserved across MAH strains, regardless of lineage or strain background. Together, these genes define a conserved genetic backbone upon which lineage-specific adaptations are superimposed. Interestingly, only three genes showed partial strain-dependent differences in essentiality despite being classified as core essential by the HMM method. These genes are involved in key metabolic and cell envelope–associated pathways, including pantothenate biosynthesis (*panC*), arabinogalactan biosynthesis (*gftB*) and the glycine cleavage system linked to one-carbon metabolism (*gcvP*). The observed variation in genetic requirement may therefore reflect subtle lineage-dependent differences in metabolic network organization or cell envelope physiology. Further experimental studies will be required to determine the mechanistic basis underlying these strain-dependent differences in essentiality.

In addition to defining a universal set of core-essential genes, our study revealed that the EA lineage-specific essential gene is annotated as a member of the major facilitator superfamily transporter family. This gene may play important roles in host adaptation and may contribute to the higher prevalence and drug resistance frequently observed in the EA lineage [45]. Resampling analysis further revealed that 88 genes were more required in MAC109, 111 genes were more required in OCU682 and 90 genes were more required in OCU683 when compared with OCU803. Genes showing reduced genetic requirement in OCU803 were enriched in pathways related to cell envelope biosynthesis, respiratory metabolism and cofactor metabolism. These included genes involved in arabinogalactan and lipid metabolism (e.g. *pimB*, *aftB* and *lmeA*), components of the respiratory chain (e.g. nuo genes and cytochrome oxidases) and enzymes involved in folate and pantothenate metabolism. This pattern suggests that OCU803 may possess alternative metabolic strategies or compensatory pathways that reduce dependency on these functions compared with other strains. This observation indicates that essentiality can diverge not only between EA and SC but also within closely related lineages. Such strain-dependent resiliency has been reported not only in *Mtb* but also in *Streptococcus pneumoniae*, where it contributes to clinically relevant phenotypes [49]. Beyond the biological insights, our study also provides a practical framework for comparing gene essentiality across several strains using Tn-Seq data. In many comparative studies, essentiality differences are often inferred directly from binary HMM classifications, which can be sensitive to differences in transposon insertion density, library saturation and sequencing depth, particularly when integrating public datasets. By combining ortholog-based pan-genome mapping with binary HMM classification and a resampling-based statistical framework, our approach enables more robust identification of conserved and lineage-dependent genetic requirements across strains. This strategy helps distinguish true biological differences in gene requirement from technical variation and may facilitate future comparative functional genomics studies across diverse bacterial populations. Considering that each MAH strain harboured more than 500 accessory genes, our results underscore the plasticity of essentiality and its modulation by the accessory genome, consistent with recent pan-genome studies in other pathogens [16,50, 51]. This plasticity poses both challenges and opportunities for drug development. From a therapeutic perspective, the limited set of core-essential genes (e.g. *pbpB*, *dprE1*, *inhA*) emphasizes the challenge of finding universally conserved targets. In particular, PBPs, the targets of β-lactams, were not included in the core-essential genes. Although this study analysed a relatively small number of strains and further experimental validation will be required, these findings suggest physiological adaptations that may influence drug susceptibility. Taken together, our results highlight both the species-specific patterns of essentiality and the need to distinguish conserved from lineage-variable targets when prioritizing therapeutic strategies.

## Supplementary material

10.1099/mgen.0.001753 Supplementary Material 1.

10.1099/mgen.0.001753 Supplementary Material 2.

## Abbreviations

COG: Clusters of Orthologous Genes
EA: East Asia
gDNA: genomic DNA
HMM: Hidden Markov Model
MAC: *Mycobacterium avium* complex
MAC-PD: *Mycobacterium avium* complex pulmonary disease
MAH: *Mycobacterium avium* subsp. *hominissuis*
*Mtb*: *Mycobacterium tuberculosis*
NCBI: National Center for Biotechnology Information
NTM-PD: pulmonary disease caused by non-tuberculous mycobacteria
OADC: oleate-albumin-dextrose-catalase
PBP: penicillin-binding protein
PGAP: Prokaryotic Genome Annotation Pipeline
SC: sequence cluster
SMRT: single-molecule real-time sequencing
Tn-Seq: transposon insertion sequencing
