# Supplementary material for: Integrated analysis of transposon insertion sequencing and pangenome reveals core and lineage-specific essential genes in Mycobacterium avium subsp. hominissuis
Source: Microb Genom. 2026 Jun 25;12(6):001753. doi: 10.1099/mgen.0.001753 (PMC13298725; doi:10.1099/mgen.0.001753)
Supplement: Supplementary Material 1. [file mgen-12-01753-s001.pdf]

Supplemental Information for:

Integrated analysis of Tn-Seq and pangenome reveals core and lineage-specific essential genes in *Mycobacterium avium* subsp. *hominissuis*

Supplementary Figures:

Supplementary Figure 1 - Flower plot illustrating the core and accessory genes among the 26 isolates.

Supplementary Figure 2 - Functional composition of core and accessory genomes based on COG classification.

Supplementary Figure 3 - *In vitro* TRANSIT classification of genes in OCU683 and MAC109 isolates.

Supplementary Figure 4 - Essential genes identified by the TRANSIT HMM method.

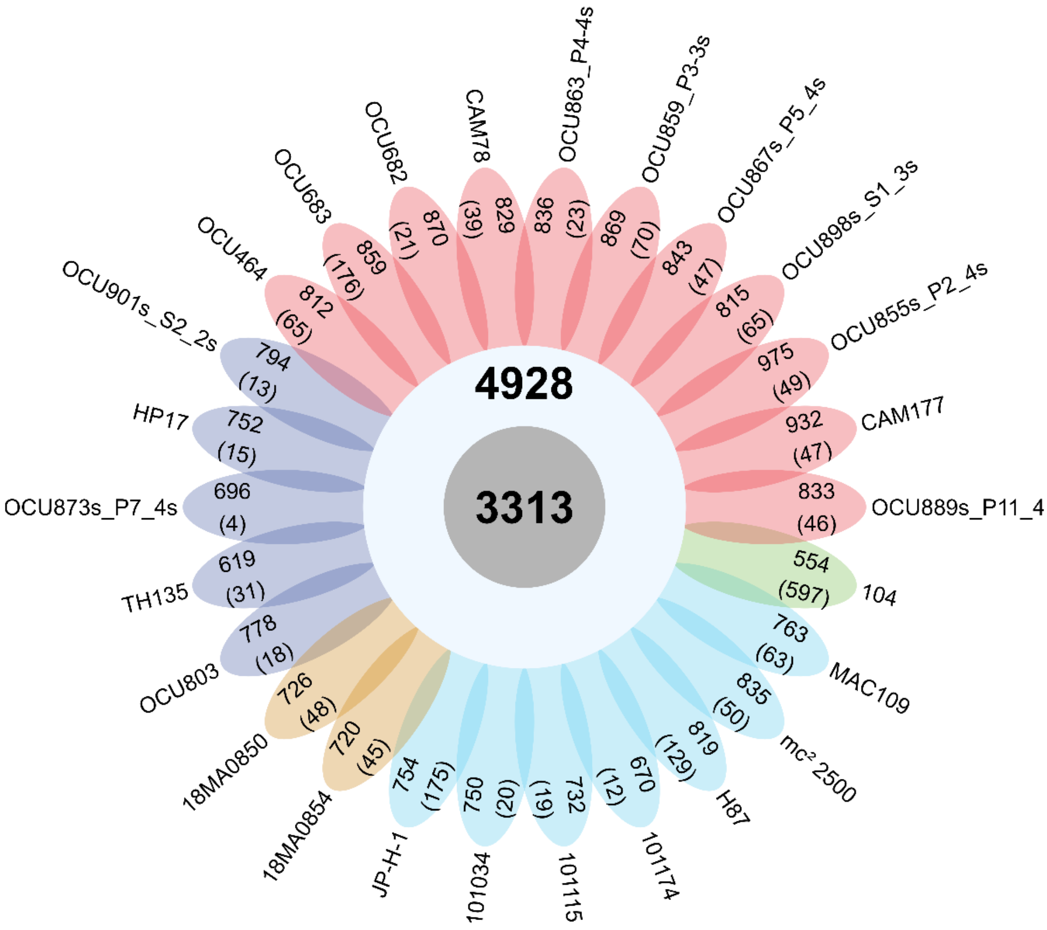

Supplementary Figure 1. Flower plot illustrating the core and accessory genes among the 26 isolates.

The center circle represents the core genes (conserved in all strains). The outer leaflets show the number of accessory genes in each isolate, with numbers in parentheses indicating strain-specific accessory genes. Leaflet colors correspond to the lineages shown in Figure 1.

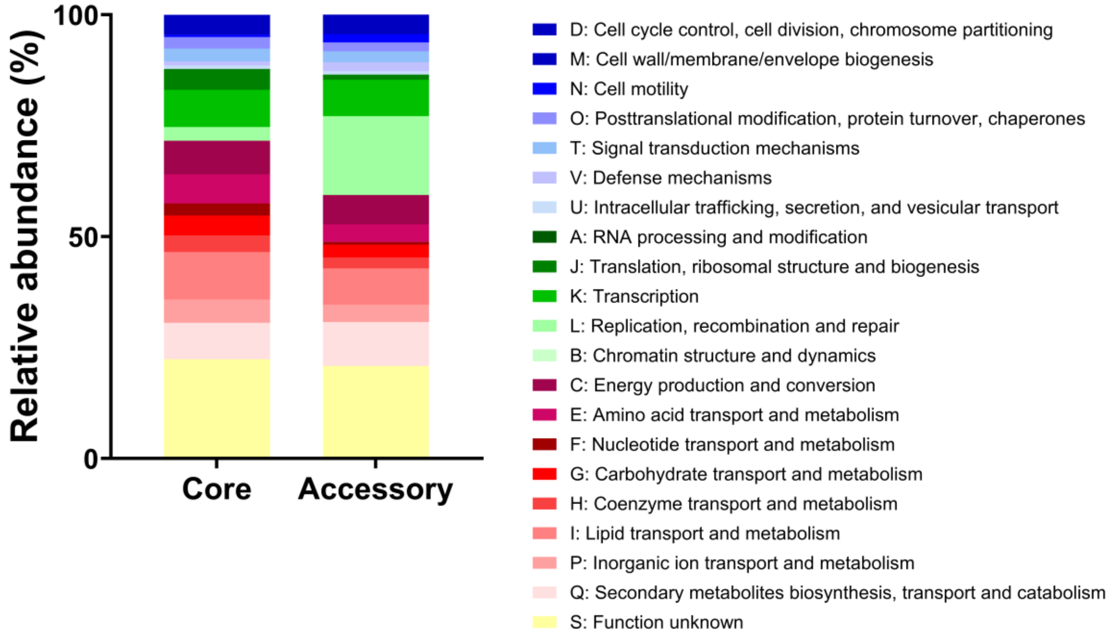

Supplementary Figure 2. Functional composition of core and accessory genomes based on COG classification.

Relative abundance of COG functional categories among core and accessory genes identified in *Mycobacterium avium* strains MAC109, OCU682, OCU683, and OCU803. Core genes were defined as orthologous genes present in all 26 analyzed strains based on Roary analysis, whereas accessory genes included soft-core, shell, and cloud genes. Each bar represents the proportional distribution of genes assigned to each COG functional category.

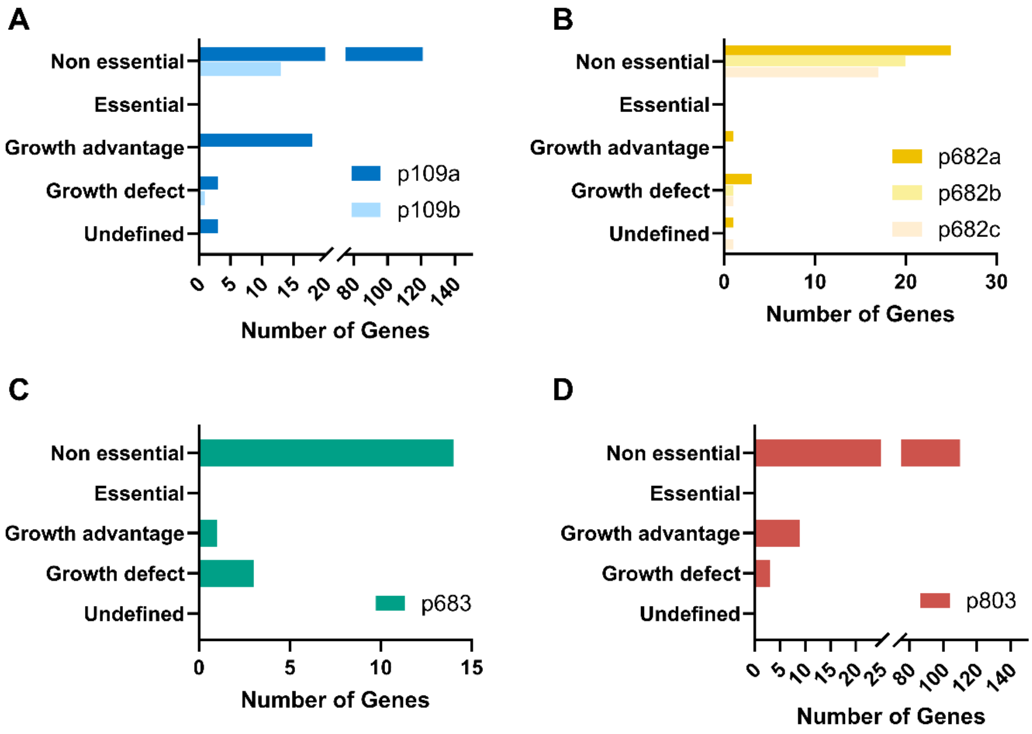

Supplementary Figure 3. *In vitro* TRANSIT classification of genes in OCU683 and MAC109 isolates.

The numbers of genes classified as non-essential, essential, growth advantage, growth defect, and undefined are shown for: A) MAC109 plasmids (p109a, p109b), B) OCU682 plasmids (p682a, p682b, p682c), C) OCU683 plasmid (p683), D) OCU803 plasmid (p803).

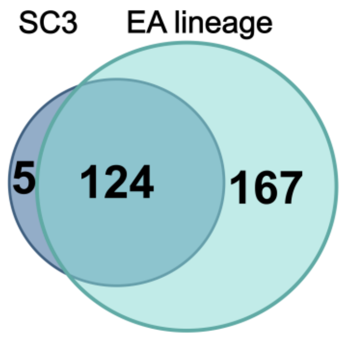

Supplementary Figure 4. Essential genes identified by the TRANSIT HMM method.

Venn diagram showing the *in vitro* essential genes identified in EA lineage strains (OCU682, OCU683, and OCU803) and the SC3 lineage strain (MAC109) based on the TRANSIT HMM analysis. Genes in the EA group represent those classified as essential in at least one EA strain (OCU682, OCU683, or OCU803) but non-essential in the SC3 strain MAC109. Low-confidence genes classified by TRANSIT were excluded from the analysis.
